# A Sweet Response to a Sour Situation: The Role of Soluble Pattern Recognition Receptors in the Innate Immune Response to Invasive *Aspergillus fumigatus* Infections

**DOI:** 10.1371/journal.ppat.1005637

**Published:** 2016-07-14

**Authors:** Stefan Bidula, Silke Schelenz

**Affiliations:** 1 Aberdeen Fungal Group, School of Medical Sciences, Institute of Medical Sciences, University of Aberdeen, Aberdeen, United Kingdom; 2 Department of Microbiology, Royal Brompton Hospital, London, United Kingdom; Geisel School of Medicine at Dartmouth, UNITED STATES

## Introduction

*Aspergillus* spp. infect around 11,000,000 patients, resulting in about 600,000 deaths per year, but these numbers are on the rise due to the emergence of antifungal-resistant strains and a lack of sensitive diagnostic tests [1].

It is increasingly acknowledged that soluble pattern recognition receptors (PRRs), such as the complement component C1q, the collectins (MBL, SP, and CL-11), PTX3, and the ficolins (ficolin-1, 2, 3 and A), are important within anti-*Aspergillus* immunity [2]. Moreover, studies have highlighted that they may be used as a possible alternative to current antifungal drugs or used in combination to increase efficacy [3].

Binding of pathogen-associated molecular patterns (PAMPs) on the pathogen surface by soluble PRRs often results in opsonisation. This enhances interactions with membrane-associated PRRs on phagocytes, such as the important β-glucan receptor Dectin-1, Toll-like receptors (TLRs), complement receptors (CR1), and Fc receptors; ultimately augmenting phagocytosis, which is essential in controlling the infection [2].

Alternatively, opsonins can promote fungal damage directly or further promote opsonisation by C3b deposition via activation of the conserved complement system [4]. There are three main arms of the complement system, which are the classical, alternative, and lectin pathways. C1q primarily activates the classical antibody-mediated pathway, whereas MBL, CL-11, and the ficolins are known to activate the lectin complement pathway via activation of the mannose-binding lectin-associated serine proteases (MASPs). However, SP-A and SP-D are not involved in complement activation, and the role of CL-11 in *Aspergillus* immunity is yet to be explored. Furthermore, PTX3 can interact with complement activators and inhibitory components to modulate all three pathways [5]. The role of each of these PRRs in anti-*Aspergillus* immunity will be discussed further.

## PTX3 Plays a Non-redundant Role in *Aspergillus fumigatus* Immunity

PTX3 is a globally expressed acute-phase protein that is synthesised locally at inflammatory sites by several cell types, particularly mononuclear phagocytes, dendritic cells (DCs), epithelial, and endothelial cells. Furthermore, PTX3 is stored within neutrophil granules containing lactoferrin and once secreted, associates with neutrophil extracellular traps (NETs), acting as a focal point for antimicrobial effector molecules [6].

PTX3 primarily functions as an opsonin in *A*. *fumigatus* immune responses, whereby it binds to galactomannan residues of dormant spores, facilitating recognition and phagocytosis [7]. PTX3 can also interact with numerous important opsonins, complement proteins, and membrane-associated PRRs to enhance antifungal immunity, including MBL, ficolin-2, C1q, Factor H, and Dectin-1, and more recently has been shown to exert its antifungal effects through TLR4/MD-2 mediated signalling [8,9]. Moreover, PTX3 can modulate all three complement pathways [5]. Current evidence indicates that PTX3 activates complement on the *Aspergillus* conidial surface and interacts with FcγRIIa, which mediates activation of the complement receptor CR3, leading to recognition and internalization of conidia [10].

There have been several human studies reporting single nucleotide polymorphisms (SNPs) in the PTX3 gene that are associated with susceptibility to *A*. *fumigatus* infections in haematopoietic stem cell and whole organ transplant patients [11,12].

In support of these findings, studies utilising PTX3 knockout mice have indicated a non-redundant role within immunity to *A*. *fumigatus* pulmonary infection [7]. Furthermore, PTX3 has been demonstrated to be protective against invasive aspergillosis (IA) in mice receiving allogeneic bone marrow transplants, in chronic granulomatous disease mice (p47^phox-/-^), and corticosteroid-treated rats [13].

## Mannose-Binding Lectin (MBL) Is Essential for Defence Against *A*. *fumigatus*

**Fig 1:**
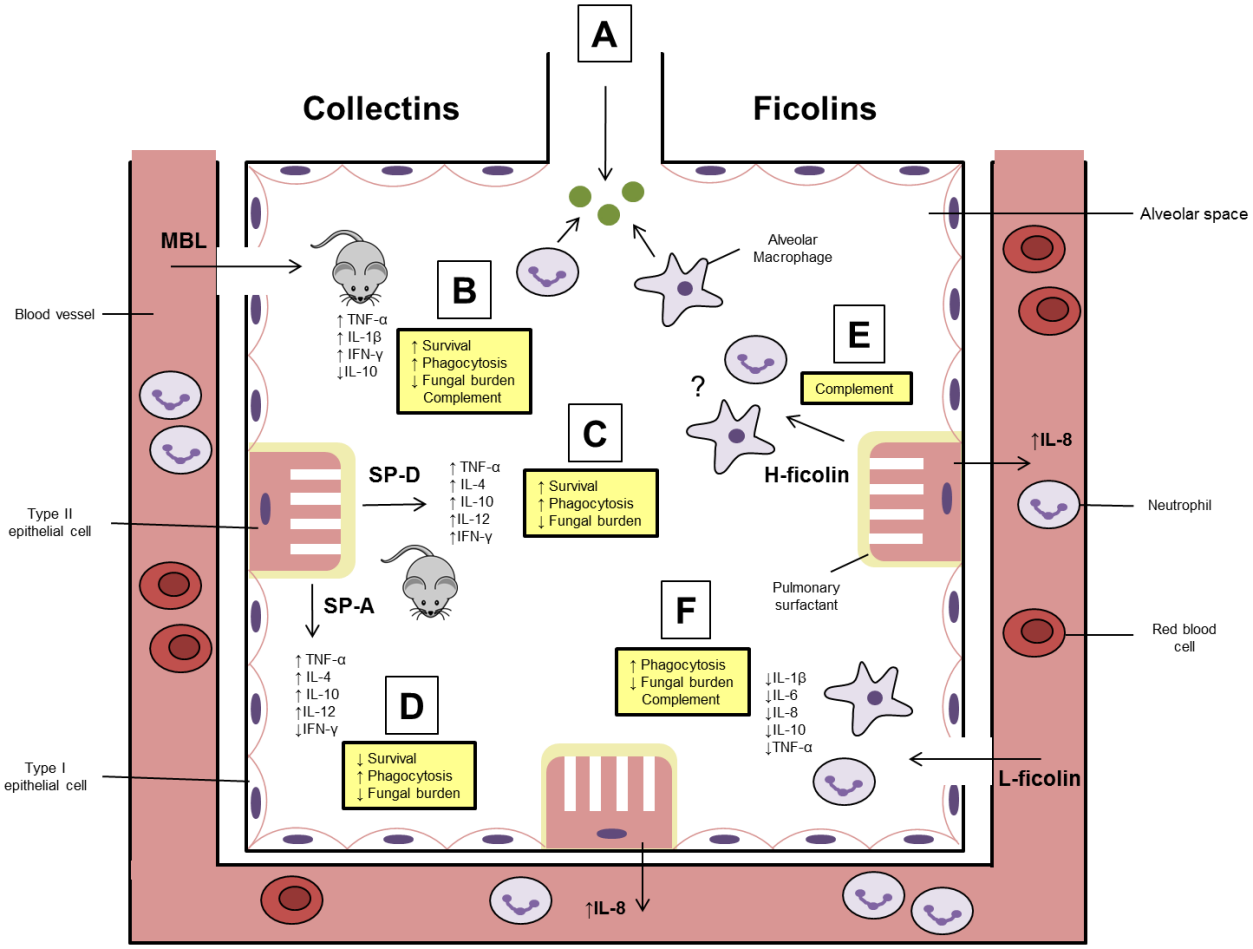
The role of serum lectins within the alveolar space. **(A)** Upon entry into the alveolar space, resident neutrophils and macrophages are essential in the recognition and effective removal of *A*. *fumigatus*. **(B)** In the event that *A*. *fumigatus* evades removal, it can germinate into a filamentous hyphal form, which causes damage to the lung epithelium and vasculature. This leads to the leakage of serum mannose-binding lectin (MBL) into the alveolar space where it interacts with *A*. *fumigatus*. It has been demonstrated in vivo that MBL is capable of modulating inflammatory cytokine production, enhancing phagocytosis, fungal killing, and survival. Moreover, MBL activates the lectin complement pathway. **(C** and **D)** The surfactant proteins (SP)-A and –D are both secreted directly into the alveolar space by type II epithelial cells. SP-A can predominantly be found within the pulmonary surfactant, whereas most of the SP-D can be found within the bronchoalveolar lavage fluid (BAL). As for MBL, SP-A and SP-D have been demonstrated to modulate cytokines, increase phagocytosis, and reduce fungal burden in vivo. However, SP-A appeared to be detrimental to survival following *A*. *fumigatus* infection, and neither are capable of activating complement. **(E)** H-ficolin is the most abundant ficolin in the serum, but it is also produced directly into the alveolar space by type II epithelial and bronchial cells. H-ficolin is capable of activating the lectin complement pathway on *A*. *fumigatus* conidia, and H-ficolin opsonised conidia promote the secretion of IL-8 from type II epithelial cells. However, the interactions of H-ficolin opsonised *A*. *fumigatus* with other cells of the immune system (neutrophils and macrophages) or in vivo (H-ficolin is a pseudogene in rodents) is unknown. **(F)** L-ficolin is found circulating in the serum but can enter the alveolar space following fungal infection. L-ficolin opsonisation has been demonstrated to lead to a reduction in inflammatory cytokine production by neutrophils and macrophages, promote IL-8 production by type II epithelial cells, increase host–fungal interactions, and activate the lectin pathway of complement. However, the in vivo function of this protein is still unknown.

MBL is one of the best characterised lectins involved in innate antifungal immunity. It is found predominantly within the serum, but, during inflammation, loss of vascular integrity can result in leakage of MBL into alveola where it can interact with *A*. *fumigatus* (Fig 1) [14]. Binding here is primarily achieved via selective and calcium-dependent binding to the carbohydrate moieties D-mannose, L-fucose, and *N*-acetylglucosamine (GlcNAc) in the *A*. *fumigatus* cell wall.

Neth et al. [15] were the first to show demonstrable binding of *A*. *fumigatus* by the MBL carbohydrate recognition domain (CRD). It wasn’t until much later that MBL was described to be protective against *Aspergillus* infection via the activation of the lectin-complement pathway on *A*. *fumigatus* conidia.

It has since been well established in humans that natural MBL deficiencies, or MBL deficiencies due to genetic polymorphisms, are significantly correlated with increased susceptibility to acute IA and chronic necrotizing pulmonary aspergillosis (CPA), respectively [16,17].

This importance has been well documented in murine models by Kaur et al., [18] in which they comprehensively demonstrated that MBL-deficiency was linked to significantly reduced phagocytosis, diminished complement activation, impaired cytokine responses, and greater mortality in a murine model of IA.

Furthermore, studies utilising serum obtained from transgenic animals have indicated that only MBL-C, and not MBL-A, can recognise *A*. *fumigatus* and is essential for complement activation [19].

Conversely, a more recent study indicated that loss of MBL in a systemic model of aspergillosis resulted in a resistant phenotype and may play a deleterious role [20], suggesting an importance within pulmonary infection rather than disseminated disease.

## Surfactant Protein-D Is an Important Initiator of the Fungal Immune Response to *A*. *fumigatus*

The roles of SP-A and SP-D in *Aspergillus* defence have been extensively studied, with SP-D exhibiting particular importance. SP-D is found in alveolar lung lining and primarily binds β-1,6-glucan in the *A*. *fumigatus* cell wall. Interestingly, SP-D can also bind *A*. *fumigatus* hyphae in a calcineurin-sensitive manner, hinting at an additional role in the later stages of infection [21].

Recognition by SP-D has been observed to augment the immune response to *Aspergillus* in vitro and in vivo. In particular, SP-D is essential in vivo, whereby it has been observed that administration of SP-D can protect immunosuppressed mice against an otherwise fatal dose of *Aspergillus*, and SP-D–deficient mice are highly susceptible to IA [22,23]. Conversely, SP-A–deficient mice become more resistant to invasive infection, indicating SP-A may even facilitate pathology [23].

However, it appears that surfactant proteins may play a greater role within allergic bronchopulmonary aspergillosis (ABPA) rather than IA. Human studies have indicated a polymorphism in the collagen region of SP-A (SP-A2) that is correlated with increased risk of ABPA and increased allergic responses, but no SNPs have so far been shown to enhance susceptibility to IA [24].

## Ficolins: The Emergence of a Novel Participant in the Host Fungal Response

We and others have recently implicated ficolins within fungal host–microbe interactions. L-ficolin and H-ficolin, in addition to rodent ficolin-A, bind avidly to *A*. *fumigatus* via a range of carbohydrate moieties, including GlcNAc, *N-*aceytlgalactosamine, D-mannose, and L-fucose [19,25–28]. Furthermore, ficolin-A also recognises the resting, swollen, and germinating morphotypes of *A*. *fumigatus*, in addition to the less pathogenic species: *A*. *flavus*, *A*. *terreus*, and *A*. *niger* [19].

Following binding to *A*. *fumigatus*, both L- and H-ficolin activate the lectin-complement pathway on *A*. *fumigatus* conidia, whereas ficolin-A was shown to play a redundant role to MBL-C [19,26,27]. Consequently, opsonisation by L-ficolin, ficolin-A, and H-ficolin has been demonstrated to enhance the phagocytosis of conidia by primary macrophages, neutrophils, and the type II epithelial cell line (A549), but it is only following interaction with the macrophages and neutrophils where significant fungal killing is observed [25,26,29].

Furthermore the inflammatory response elicited by ficolin-opsonised conidia is dependent upon the cell type involved. Following cell challenge with ficolin-opsonised conidia, a MAPK-dependent increase in IL-8 production was observed from epithelial cells, whereas down-regulation of IL-1β, IL-6, IL-8, IL-10, and TNF-α production was observed from macrophages and neutrophils via currently uncharacterized mechanisms [25,26,29]. These observations have raised some interesting questions; however, the implications of ficolins in disease models have yet to be elucidated, and our understanding of the role of ficolins in antifungal immunity are in their infant stages.

## Diagnostic and Therapeutic Potential of Soluble PRRs

Antifungal drug resistance and a lack of conclusive diagnostics are two of the major challenges limiting the cure of aspergillosis, and many opsonins demonstrate therapeutic potential.

It has been demonstrated that administration of recombinant MBL is protective in a murine model of invasive *A*. *fumigatus* infection and can significantly reduce mortality [18]. The therapeutic potential of SP-D has also been explored in mice, and administration of native and recombinant SP-D is associated with decreased fungal burden in the lungs and increased levels of antifungal IFN-γ in IA [30].

Administration of recombinant PTX3 can ameliorate infection and increase survival in a pulmonary model of *A*. *fumigatus* infection in mice [13]. An interesting caveat of PTX3 is its ability to have an additive effect on the efficacy of commonly used antifungals such as ambisome and voriconazole [3]. Importantly, in combination with PTX3, the antifungal dose could be lowered whilst maintaining efficacy, which could lead to the reduced risk of drug-related side effects. This would be especially beneficial for severely immunocompromised patients [3].

Unlike MBL, PTX3, and SP-D, the therapeutic potential of ficolins is yet to be explored. Unsurprisingly, H-ficolin BAL concentrations are increased during *A*. *fumigatus* infection, but as L-ficolin is not produced directly in the lung, it was hypothesised that it enters the alveolar space following vascular damage and may be useful as a diagnostic marker in combination with other fungal specific markers such as galactomannan [25,26]. As ficolins have been observed to dampen pro-inflammatory cytokine production by phagocytic cells, it could be hypothesised that they may have the potential to be exploited therapeutically [25,29].

To date, there has been no indication that soluble PRRs can be exploited for their diagnostic potential, albeit the presence of PRRs such as L-ficolin and MBL in the lung during inflammation and infection highlights the necessity to investigate soluble PRRs as potential diagnostic tools. Moreover, further larger-scale clinical trials are needed to assess the full diagnostic potential of ficolins and other PRRs in combination with current fungal and host biomarkers in order to evaluate their role in diagnostics and possible impact on patient outcomes.
